# Supplementary material for: Prevalence and factors associated with psychological distress among key populations in Nigeria
Source: PLoS One. 2024 Apr 16;19(4):e0300901. doi: 10.1371/journal.pone.0300901 (PMC11020377; doi:10.1371/journal.pone.0300901)
Supplement: Attachment — Submitted filename: PONE-D-23-38088_R1_GA_edits.docx [file pone.0300901.s002.docx]

PLOS ONE

Prevalence and Factors Associated with Psychological Distress among Key Populations in Nigeria

--Manuscript Draft--

| Manuscript Number: | PONE-D-23-38088 |
| --- | --- |
| Article Type: | Research Article |
| Full Title: | Prevalence and Factors Associated with Psychological Distress among Key Populations in Nigeria |
| Short Title: | Prevalence and Factors Associated with Psychological Distress among Key Populations in Nigeria |
| Corresponding Author: | Olaniyi Felix Sanni, PhD  Heartland Alliance International Nigeria Abuja, NIGERIA |
| Keywords: | Mental health, Psychological stress, Key population, One stop shop, Disparities |
| Abstract: | Background: Stigmatisation and discrimination within healthcare settings deter key populations (KPs) from seeking mental health and psychosocial support (MHPS). Consequently, understanding the prevalence and contributing factors of Psychological Distress (PD) among these groups is crucial.  Method: This is a cross-sectional study focused on KPs, including Female Sex Workers (FSW), Men who have Sex with Men (MSM), and People Who Inject Drugs (PWID) enrolled in Heartland Alliance LTD/GTE across 17 One-Stop Shops (OSS) in six states of Nigeria. Data were extracted from the databases of the OSS. PD was assessed using the Mental Health Screening Form III (MHSF-III), and data were analysed using IBM-SPSS version 28.  Results: The prevalence of PD among the KPs was 9.7%. Higher rates were observed among FSWs (12.0%). Akwa Ibom and Lagos states recorded alarming prevalences at 53.6% and 31.6%. HIV- positive KPs had a prevalence of 11.6%, while those with histories of alcohol/drug use and gender-based violence showed nearly universal prevalence at 97.7% and 99.0%. Adjusted odds ratios revealed that state of residence, alcohol/drug use, and gender-based violence were potent predictors of PD. Conclusion: The study highlights the critical need for targeted mental health interventions among KPs in Nigeria, primarily focusing on those with a history of substance abuse and gender-based violence. Despite universal brief interventions, the proportion enrolled in further Psychosocial support indicates a need to improve mental health service utilisation among the KPs in Nigeria. |
| Order of Authors: | Olaniyi Felix Sanni, PhD |
|  | Roger Abang, MBBCH, MSc |
|  | Godwin Emmanuel, MBBS, MSc |
|  | Paul Umoh |
|  | Abiye Kalaiwo |
|  | Nanribet Mwoltu |
|  | Paul Amechi, HND, PGD, MHIM |
|  | Bartholomew Ochonye, BSC, MSc, PHD |
|  | Olugbemi Motilewa |
| Additional Information: |  |
| Question | Response |
| Financial Disclosure  Enter a financial disclosure statement that | Yes |

| describes the sources of funding for the work included in this submission. Review the [submission guidelines](http://journals.plos.org/plosone/s/submission-guidelines#loc-financial-disclosure-statement) for detailed requirements. View published research articles from [*PLOS ONE*](https://journals.plos.org/plosone/) for specific examples.  This statement is required for submission and will appear in the published article if the submission is accepted. Please make sure it is accurate. |  |
| --- | --- |
| Funded studies  Enter a statement with the following details:   - Initials of the authors who received each award - Grant numbers awarded to each author - The full name of each funder - URL of each funder website - Did the sponsors or funders play any role in the study design, data collection and analysis, decision to publish, or preparation of the manuscript? |  |
| Did you receive funding for this work? |  |
| Please add funding details.  as follow-up to "Financial Disclosure  Enter a financial disclosure statement that describes the sources of funding for the work included in this submission. Review the [submission guidelines](http://journals.plos.org/plosone/s/submission-guidelines#loc-financial-disclosure-statement) for detailed requirements. View published research articles from [*PLOS ONE*](https://journals.plos.org/plosone/) for specific examples.  This statement is required for submission and will appear in the published article if the submission is accepted. Please make sure it is accurate. | Funding and data used for analysis was made available by Heartland alliance LTD/GTE (HALG) |

| Funded studies  Enter a statement with the following details:   - Initials of the authors who received each award - Grant numbers awarded to each author - The full name of each funder - URL of each funder website - Did the sponsors or funders play any role in the study design, data collection and analysis, decision to publish, or preparation of the manuscript? |  |
| --- | --- |
| Did you receive funding for this work?" |  |
| Please select the country of your main research funder (please select carefully as in some cases this is used in fee calculation).  as follow-up to "Financial Disclosure  Enter a financial disclosure statement that describes the sources of funding for the work included in this submission. Review the [submission guidelines](http://journals.plos.org/plosone/s/submission-guidelines#loc-financial-disclosure-statement) for detailed requirements. View published research articles from [*PLOS ONE*](https://journals.plos.org/plosone/) for specific examples.  This statement is required for submission and will appear in the published article if the submission is accepted. Please make sure it is accurate. | NIGERIA - NG |
| Funded studies  Enter a statement with the following details:   - Initials of the authors who received each award - Grant numbers awarded to each author - The full name of each funder - URL of each funder website - Did the sponsors or funders play any role in the study design, data collection and analysis, decision to publish, or preparation of the manuscript? |  |
| Did you receive funding for this work?" |  |
| Competing Interests | The authors have declared that no competing interests exist. |

| Use the instructions below to enter a competing interest statement for this submission. On behalf of all authors, disclose any [competing interests](http://journals.plos.org/plosone/s/competing-interests) that could be perceived to bias this work—acknowledging all financial support and any other relevant financial or non- financial competing interests.  This statement is required for submission and will appear in the published article if the submission is accepted. Please make sure it is accurate and that any funding sources listed in your Funding Information later in the submission form are also declared in your Financial Disclosure statement.  View published research articles from  [*PLOS ONE*](http://journals.plos.org/plosone/) for specific examples. |  |
| --- | --- |
| NO authors have competing interests  Enter: *The authors have declared that no competing interests exist.*  Authors with competing interests  Enter competing interest details beginning with this statement:  *I have read the journal's policy and the authors of this manuscript have the following competing interests: [insert competing interests here]* |  |
| * typeset |  |
| Ethics Statement  Enter an ethics statement for this submission. This statement is required if the study involved:   - Human participants - Human specimens or tissue - Vertebrate animals or cephalopods - Vertebrate embryos or tissues | Approval certificate letter for this research was gotten from university of Uyo teaching hospital, Uyo institutional health research ethical committee (IHREC). A copy of this letter is uploaded alongside the manuscript and cover letter. Additionally, both written and verbal consent were obtained from the study participants. The consent form was written in English and translated into Pidgin English, a general language that all Nigerians understand. For those who could not read, the protocol was read to them until they understood, and those who agreed to participate in the study were made to thumbprint the consent form. |

| - Field research   Write "N/A" if the submission does not require an ethics statement.  General guidance is provided below. Consult the [submission guidelines](http://journals.plos.org/plosone/s/submission-guidelines#loc-guidelines-for-specific-study-types) for detailed instructions. Make sure that all information entered here is included in the Methods section of the manuscript. |  |
| --- | --- |

| Format for specific study types  Human Subject Research (involving human participants and/or tissue)   - Give the name of the institutional review board or ethics committee that approved the study - Include the approval number and/or a statement indicating approval of this research - Indicate the form of consent obtained (written/oral) or the reason that consent was not obtained (e.g. the data were analyzed anonymously)   Animal Research (involving vertebrate animals, embryos or tissues)   - Provide the name of the Institutional Animal Care and Use Committee (IACUC) or other relevant ethics board that reviewed the study protocol, and indicate whether they approved this research or granted a formal waiver of ethical approval - Include an approval number if one was obtained - If the study involved *non-human primates*, add *additional details* about animal welfare and steps taken to ameliorate suffering - If anesthesia, euthanasia, or any kind of animal sacrifice is part of the study, include briefly which substances and/or methods were applied   Field Research  Include the following details if this study involves the collection of plant, animal, or other materials from a natural setting:   - Field permit number - Name of the institution or relevant body that granted permission |  |
| --- | --- |
|  |  |
| Data Availability  Authors are required to make all data underlying the findings described fully available, without restriction, and from the time of publication. PLOS allows rare exceptions to address legal and ethical concerns. See the [PLOS Data Policy](http://journals.plos.org/plosone/s/data-availability) and [FAQ](http://journals.plos.org/plosone/s/data-availability#loc-faqs-for-data-policy) for detailed information. | Yes - all data are fully available without restriction |

| A Data Availability Statement describing where the data can be found is required at submission. Your answers to this question constitute the Data Availability Statement and will be published in the article, if accepted. |  |
| --- | --- |
| Important: Stating ‘data available on request from the author’ is not sufficient. If your data are only available upon request, select ‘No’ for the first question and explain your exceptional situation in the text box. |  |
| Do the authors confirm that all data underlying the findings described in their manuscript are fully available without restriction? |  |
| Describe where the data may be found in full sentences. If you are copying our sample text, replace any instances of XXX with the appropriate details.   - If the data are held or will be held in a public repository, include URLs, accession numbers or DOIs. If this information will only be available after acceptance, indicate this by ticking the box below. For example: *All XXX files are available from the XXX database (accession number(s) XXX, XXX.*). - If the data are all contained within the manuscript and/or Supporting Information files, enter the following: *All relevant data are within the manuscript and its Supporting Information files.* - If neither of these applies but you are able to provide details of access elsewhere, with or without limitations, please do so. For example:   *Data cannot be shared publicly because of [XXX]. Data are available from the XXX Institutional Data Access / Ethics Committee (contact via XXX) for researchers who meet the criteria for access to confidential data.*  *The data underlying the results presented in the study are available from (include the name of the third party* | All relevant data are within the manuscript and its Supporting Information files |

| *and contact information or URL).*   - This text is appropriate if the data are owned by a third party and authors do not have permission to share the data.   * typeset |  |
| --- | --- |
| Additional data availability information: |  |

Manuscript	[Click here to access/download;Manuscript;PLOS ONE -](https://www.editorialmanager.com/pone/download.aspx?id=34713228&guid=2cf521dc-6923-4771-96b2-bde1758d6b8f&scheme=1)[<https://www.editorialmanager.com/pone/download.aspx?id=34713228&guid=2cf521dc-6923-4771-96b2-bde1758d6b8f&scheme=1> PYSCHOLOGICAL DISTRESS.docx](https://www.editorialmanager.com/pone/download.aspx?id=34713228&guid=2cf521dc-6923-4771-96b2-bde1758d6b8f&scheme=1)

**Prevalence and Factors Associated with Psychological Distress**

**among Key Populations in Nigeria**

Authors' information:

1. Olaniyi Felix Sanni – Research and Development Department, Heartland Alliance, Abuja, Nigeria, [sfelix@heartlandalliancenigeria.org,](mailto:sfelix@heartlandalliancenigeria.org) BSc, MSc, MPH, PhD – Corresponding Author
2. Roger Abang- Heartland Alliance Nigeria, MBBCH, MSc
3. Godwin Emmanuel-Heartland Alliance Nigeria, MBBS, MSc
4. Paul Umoh- Heartland Alliance Nigeria, BA, MSW
5. Abiye Kalaiwo-USAID Nigeria, MBBS, MPH
6. Nanribet Mwoltu- Heartland Alliance Nigeria Bsc, Msc,
7. Paul Amechi- Heartland Alliance Nigeria HND, PGD, MHIM
8. Bartholomew Ochonye -Heartland Alliance Nigeria, Bsc, MSc., PHD
9. Olugbemi Motilewa- University of uyo Teaching hospital MBBS,MPH,FWACP

**Abstract**

**Background**: Stigmatization and discrimination within healthcare settings deter key populations (KPs) from seeking mental health and psychosocial support (MHPS). Consequently, understanding the prevalence and contributing factors of Psychological Distress (PD) among these groups is crucial.

**Method**: This is a cross-sectional study focused on KPs, including Female Sex Workers (FSW), Men who have Sex with Men (MSM), and People Who Inject Drugs (PWID) enrolled in Heartland Alliance LTD/GTE across 17 One-Stop Shops (OSS) in six states of Nigeria. Data were extracted from the databases of the OSS. PD was assessed using the Mental Health Screening Form III (MHSF-III), and data were analyzed using IBM-SPSS version 28.

**Results**: The prevalence of PD among the KPs was 9.7%. Higher rates were observed among FSWs (12.0%). Akwa Ibom and Lagos states recorded alarming prevalences at 53.6% and 31.6%. HIV- positive KPs had a prevalence of 11.6%, while those with histories of alcohol/drug use and gender-based violence showed nearly universal prevalence at 97.7% and 99.0%. Adjusted odds ratios revealed that state of residence, alcohol/drug use, and gender- based violence were potent predictors of PD.

**Conclusion**: The study highlights the critical need for targeted mental health interventions among KPs in Nigeria, primarily focusing on those with a history of substance abuse and gender-based violence. Despite universal brief interventions, the proportion enrolled in further Psychosocial support indicates a need to improve mental health service utilization among the KPs in Nigeria.

**Keywords:** Mental health, Psychological stress, Key population, One stop shop, Disparities

# INTRODUCTION

Mental and Psychosocial well-being are fundamental aspects of human health, influencing overall quality of life, resilience, and the ability to thrive [1]. Among marginalized and vulnerable populations, such as female sex workers (FSW), men who have sex with men (MSM), people who inject drugs (PWID), and transgender individuals, the challenges to mental health are often exacerbated by stigma, discrimination, and marginalization [2].

Nigeria, a diverse and populous nation, is home to various key populations (KPs) facing distinct challenges related to their identities and activities. Sex workers often confront pervasive stigma, violence, and discrimination, contributing to mental health issues such as depression, anxiety, and post-traumatic stress disorder (PTSD) [3]. MSM encounter legal and social challenges, including criminalization and discrimination, resulting in significant mental distress and substance abuse [4]. In addition to battling addiction, PWID Transgender individuals grapple with high levels of discrimination, violence, and a lack of understanding, resulting in pronounced mental health disparities [5].

With the One-Stop-Shops' (OSS) purview, mental health and psychological support services (MHPSS) take on various forms designed to address the mental health challenges specific to KPs [6]. Counselling services are at the core of MHPSS, providing a safe, confidential space for individuals to discuss their mental health concerns and receive guidance and support from trained professionals. Group therapy sessions foster community and solidarity among KPs, facilitating open dialogue and reducing feelings of isolation [6]. Many OSS prioritize mental health education as a preventive measure to increase awareness of mental health issues, combat stigma, and promote self-care practices [7]. Additionally, these centres are crucial in connecting KPs with external mental health professionals and facilities when specialized interventions are required, ensuring comprehensive care is accessible [7].

Despite the noble objectives of OSS, several challenges hinder the effectiveness of MHPSS delivery. Stigmatization and discrimination within healthcare settings continue to deter KPs from seeking MHPSS, necessitating comprehensive training of healthcare providers in cultural competency and sensitivity. Limited funding and resources pose significant barriers to the availability and quality of MHPSS, emphasizing the need for increased funding for mental health service's legal framework includes provisions that criminalize the activities of KPs, creating a climate of fear and hindering access to healthcare services without fear of legal

repercussions. Advocacy and legal reform are imperative to safeguard the rights of these individuals and ensure their access to healthcare. Furthermore, disparities in MHPSS availability persist between urban and rural areas in Nigeria, necessitating efforts to establish equitable access to these services across all country regions. This paper examines the prevalence and factors of psychological distress (PD) among KPs enrolled in OSS in Nigeria.

# METHODOLOGY

**Participants**

The study included 22,310 Key Populations (KPs) who used HALG services between September 2022 and July 2023, a period of ten months. On average, participants were 31 years old, with a standard deviation **7.3**. There were 48.5% males and 51.5% females among the total subjects, representing three (3) separate KP groups (see Table 1). The purposive sample technique was used to ensure that the following requirements were met:

## Inclusion Criteria:

- Must identify as a KP.
- Must be aged 18 years and above.
- Must be accessing HALG services

## Exclusion Criteria:

- Non-KPs.
- Not assessing HALG services.

**Study design**

This research utilized a cross-sectional study design to investigate the prevalence and factors associated with psychological distress among KPs in Nigeria. The study focused on various groups such as FSW, MSM, and PWID. These groups are all participants in the Heartland Alliance LTD/GTE One Stop Shops program, which operates in multiple states across Nigeria.

Data for this study were extracted from the HA LTD/GTE OSS databases across the selected states in Nigeria. The aim was to provide a thorough understanding of psychological distress prevalence and its associated factors among KPs. Data were extracted from 17 OSS in six Nigerian states: Lagos, Bayelsa, Cross River, Akwa Ibom, Jigawa, and Niger. The data was assessed for analysis in August 2023

The assessment tool used by Heartland Alliance LTD/GTE for evaluating mental health was the Mental Health Screening Form III (MHSF-III). It was developed to screen for possible mental health problems, particularly in substance abuse treatment [8]. This questionnaire consists of "18 "yes" and "no" questions related to the respondent's history. It has a maximum score of 18, with question 6 having two parts [8]. The MHSF-III is scored by totaling the "yes" responses (1 point each) for a maximum score of 17. It includes questions 3 through 17 [8]. If specific questions yield positive responses, seeking consultation with a mental health specialist for further evaluation is advisable. The form also provides a total score, facilitating research and evaluation of the relationship between mental health and substance dependence in the program. Research has shown that most MHSF-III items perform well for screening purposes, providing evidence of their validity [8].

**Data Analysis**

Data were cleaned before being imported to a spreadsheet and analyzed with IBM-SPSS version 28. To summarize the demographic and health metric data, descriptive statistics were used. Logistic regression analysis was used to determine any correlations between demographics and PD prevalence. The impact of health measures on PD results was investigated using multiple regression analysis. Chi-squared tests investigated any links between PD screening findings and treatment outcomes.

**Ethical consideration**

Heartland Alliance's authorized KP-CARE 1 ethical clearance was used for project implementation research. Per HALG's authorization to access KP's data, this clearance was changed to maintain high data protection and security standards. To protect participant confidentiality and privacy, strict steps were implemented to anonymize and securely preserve the obtained data. Additionally, both written and verbal consent were obtained from the study participants. The consent form was written in English and translated into Pidgin English, a general language that all Nigerians understand. For those who could not read, the protocol was read to them until they understood, and those who agreed to participate in the study were made to thumbprint the consent form.

# RESULTS

**Prevalence and significance of psychological distress (PD) by Sociodemographic of KP**

The prevalence of PD among KPs across different states in Nigeria is illustrated in Table 1. Of the 22310 KPs, the prevalence of PD was 9.7% (2159 KPs). PD was higher among females (12.1%) than males (7.1%). PD was highest among FSW (12.0%), followed by PWID (8.5) and MSM (7.5%) in the key population. Participants aged 41-50 (14.6%) exhibited the highest PD compared to others in this category. Akwa Ibom (53.6%) and Lagos state (31.6%) recorded the highest prevalence of PD compared to other states. PD was higher in respondents who tested positive for HIV (11.6%) than those who tested Negative (1.4%). PD was more prevalent among KPs with a history of alcohol and drug use (97.7%) as well as those with a history of gender-based violence (99.0%), P<0.001.

**Table 1: Socio-demographics Characteristics and Prevalence of PD among KPs enrolled in HA LTD/GTE OSS across Nigeria**

| **Variable** | **Frequency (%)** | **PD Prevalence (%)** | **X^2^ (P-value)** |
| --- | --- | --- | --- |
|  | **(n= 22310)** | **(n=2159)** |  |
| **Overall** | 22310 | 2159 (9.7%) |  |
| Male | 10813 (48.5) | 773 (7.1) | 153.471 |
| **Gender**  Female | 11497 (51.5) | 1386 (12.1) | (<0.001*) |
| MSM | 8273 (37.1) | 620 (7.5) |  |

**Key**

| **Population type** | PWID | 4118 (18.5) | 352 (8.5) |
| --- | --- | --- | --- |
|  | ≤ 20 | 938 (4.2) | 119 (12.7) |
|  | 21-30 | 11325 (50.8) | 953 (8.4) |
| **Age Category** | 31-40 | 7670 (34.4) | 747 (9.7) |
|  | 41-50 | 2073 (9.3) | 303 (14.6) |
|  | 50 + | 304 (1.4) | 37 (12.2) |
|  | Bayelsa | 1939 (8.7) | 9 (0.5) |
| **State** | Akwa Ibom | 1911 (8.6) | 1025 (53.6) |
|  | Cross River | 8069 (36.2) | 177 (2.2) |

FSW	9919 (44.5)	1187 (12.0)

110.607 (<0.001*)

90.417 (<0.001*)

6611.479 (<0.001*)

|  | Jigawa | 2833 (12.7) | 0 (0.0) |  |
| --- | --- | --- | --- | --- |
|  | Lagos | 2222 (10.0) | 702 (31.6) |  |
|  | Niger | 5336 (23.9) | 246 (4.6) |  |
|  | Negative | 4129 (18.5) | 56 (1.4) | 401.355 |
| **HIV Status** | Positive | 18181(81.5) | 2103 (11.6) | (<0.001*) |

**Alcohol and**

No	22180 (99.4%)	2032 (9.2)	1158.897

**Drug use**

Yes	130 (0.6)	127 (97.7)

(<0.001*)

**Gender-Based**

No	22117 (99.1)	1968 (8.9)	1775.627

**Violence**

Yes	193 (0.9)	191 (99.0)

(<0.001*)

* Statistical significance (*P*≤0.05)

**Association between Sociodemographic Factors and Psychological Distress among KP's enrolled in OSS.**

Table 2 shows that female KPs had a higher prevalence at 12.1%. The OR was 1.78 (95% CI: 1.62-1.95, P<0.001). However, upon adjustment for other variables, the OR dropped to 0.82 (95% CI: 0.45-1.51) and was no longer statistically significant (p=0.527). MSM had a 7.5% MPHD prevalence. FSW had a 12.0% prevalence and an OR of 1.68 (95% CI: 1.52-1.86, p<0.001). However, the adjusted odds ratio was 1.52 (95% CI: 0.76-3.05, p=0.240), becoming non-significant. PWID had an 8.5% prevalence with an OR of 1.15 (95% CI: 1.01-1.32, p=0.040). Interestingly, the AOR increased to 1.95 (95% CI: 0.60-0.98, p=0.015), becoming statistically significant. When other variables are accounted for, age was not a robust predictor for PD.

Akwa Ibom showed a high prevalence of 53.6% with an OR of 24.09 (95% CI: 12.10-48.47, p<0.001) and an AOR of 27.09 (95% CI: 17.06-67.84, p<0.001), both highly significant than Bayelsa. HIV- positive KPs had an 11.6% prevalence of PD. The OR was significantly high at

9.513 (95% CI: 7.280-12.432, p<0.001). However, after adjustment, the OR plummeted to 0.74 (95% CI: 0.36-1.55) and was no longer statistically significant.

The prevalence was a staggering 97.7% with an OR of 19.75 (95% CI: 13.47-32.07, p<0.001) and an adjusted odds ratio of 21.83 (95% CI: 15.13-56.83, p<0.001), both being statistically significant. Lastly, in the case of gender-based violence, the prevalence was nearly universal at 99.0%. The OR was 17.759 (95% CI: 14.59-39.93, p<0.001), and the adjusted OR was even higher at 25.70 (95% CI: 17.10-38.73, p<0.001), both indicating strong statistical significance.

**Table 2: Association between Sociodemographic Factors and PD among KP's enrolled in OSS.**

**Variable**

**PD**

**Prevalence (%)**

**Unadjusted	Adjusted**

**OR (95% CL)	P value	OR (95% CL)	P value**

**Gender**

| Male | 773 (7.1) | Ref | - | Ref | - |
| --- | --- | --- | --- | --- | --- |
| Female | 1386 (12.1) | 1.78 (1.62-1.95) | <0.001* | 0.82 (0.45-1.51) | 0.527 |
| **Key Population** | | | | | |
| MSM | 620 (7.5) | Ref | - | Ref | - |
| FSW | 1187 (12.0) | 1.68 (1.52-1.86) | <0.001* | 1.52 (0.76-3. 05) | 0.240 |
| PWID | 352 (8.5) | 1.15 (1.01-1.32) | 0.040* | 1.95 (0.60-0.98) | 0.015 |
| **Age Category** |  |  |  |  |  |
| ≤ 20 | 119 (12.7) | 1.05 (0.71-1.56) | 0.814 | 4.64 (1.05-20.64) | 0.44 |
| 21-30 | 953 (8.4) | 0.66 (0.47-0.94) | 0.021* | 2.87 (0.72-11.44) | 0.136 |
| 31-40 | 747 (9.7) | 0.78 (0.55-1.11) | 0.164 | 2.22 (0.56-8.86) | 0.259 |
| 41-50 | 303 (14.6) | 1.24 (0.86-1.78) | 0.256 | 3.08 (0.75-12.67) | 0.119 |
| 50 +  **State** | 37 (12.2) | Ref | - | - | - |
| Bayelsa | 9 (0.5) | Ref | - | Ref | - |
| Akwa Ibom | 1025 (53.6) | 24.09 (12.10-48.47) | <0.001* | 27.09 (17.06-67.84) | <0.001* |

| Cross River | 177 (2.2) | 4.81 (128.10-480.47) | <0.001* | 13.94 (9.82-27.58) | <0.001* |
| --- | --- | --- | --- | --- | --- |
| Jigawa | 0 (0.0) | - | - | - | - |
| Lagos | 702 (31.6) | 29.04 (14.14-91.80) | <0.001* | 39.89 (26.36-93.34) | <0.001* |
| Niger | 246 (4.6) | 10.36 (5.32-20.19) | <0.001* | 13.08 (11.6-326.95) | <0.001* |
| **HIV Status** |  |  |  |  |  |
| Negative | 56 (1.4) | Ref | - | Ref | - |
| Positive | 2103 (11.6) | 9.513 (7.280-12.432) | <0.001* | 0.74 (0.36-1.55) | <0.001* |
| **Alcohol and Drug use** | | | | | |
| No | 2032 (9.2) | Ref | - | Ref | - |
| Yes | 127 (97.7) | 19.75 (13.47-32.07) | <0.001* | 21.83 (15.13-56.83) | <0.001* |
| **Gender-Based Violence** | | | | | |
| No | 1968 (8.9) | Ref | - | Ref | - |
| Yes | 191 (99.0) | 17.759 (14.59-39.93) | <0.001* | 25.70 (17.10-38.73) | <0.001* |

* Statistical significance (p 0.05)

**KP's enrolled for MHPSS Intervention.**

All Participants (100%) were given brief intervention, and 1595 of 2159 (73.8%) were referred for further psychological intervention after the brief intervention. Figure 1 shows that 752 (47.1%) of 1595 were referred for further psychological intervention and eventually enrolled for interventions.

**Types of intervention received by KP with PD**

Figure 2 illustrates different types of mental and psychological intervention services registered by participants in the intervention program. The services with the highest proportion were psychoeducation (21.20%), followed by coping skills training (17.70%) and motivational enhancement (12.90%).

**Characteristics of KPs with PD enrolled for interventions.**

The characteristics of Participants with PD were assessed in Table 3. The in-person session was conducted for 72.7% of those enrolled in the intervention, 24.6% were counselled virtually, and 2.7% in groups in the community. Many of the participants attended only one session (74.0%) in comparison to those who added two sessions (25.5%) and three sessions (0.5%), respectively. More than half of the respondents (68.3%) had good insight, 23.0% had a fair level, and 8.6% lacked insights. The confirmation outcomes show that 86.6% were mild, 12.6% were moderate, and 0.5% were severe.

**Table 3 Characteristics of KPs with PD enrolled for interventions.**

**Enrolled KP's with**

**Parameter	Variable**

**Psychological distress % (n= 752)**

Group/community	20 (2.7)

**Session Modality**	In-person	547 (72.7)

| Virtual | 185 (24.6) |
| --- | --- |
| 1 | 556 (74.0) |
| 2 | 192 (25.5) |
| 3 | 4 (0.5) |
| Fair | 173 (23.0) |
| Good | 514 (68.3) |
| Lacking | 65 (8.6) |
| Mild | 653 (86.8) |
| Moderate | 95 (12.6) |
| Severe | 4 (0.5) |

**Number of sessions**

**Current Level of Insight**

**Confirmation Outcome**

# DISCUSSION

This study examines the prevalence of Mental health and psychological well-being issues and the types of interventions administered among KPs in Nigeria. Mental health is increasingly recognized as a critical component of individual health. According to the World Health Organisation [9], mental health is an essential component of health, a state of physical, mental, social, and spiritual well-being and not merely the absence of disease or infirmity. On this premise, it is critical that the government promote, protect, and restore mental health since it may have significant consequences for the nation's progress due to disease, disability, and death among its people [10]. Nigerian society has numerous unfavourable attitudes and false notions about the mentally sick. Stigma is a huge issue in the country, causing people to be hesitant to seek treatment because they are embarrassed and fearful of prejudice and discrimination [10].

**Prevalence and significance of psychological distress by Sociodemographic of KP**

The study found varying levels of psychological distress among KPs in different states of Nigeria. This variation can be attributed to several factors, including sociodemographic characteristics and regional disparities in access to mental health services. Higher levels of psychological distress suggest a pressing need for targeted mental health interventions for KPs. A study by [11] showed that social determinants, including gender and region, often influence psychological distress. Psychological distress was more prevalent among females (12.1%) than males (7.1%) among KPs. This gender disparity could be due to unique challenges and vulnerabilities female KPs face, such as sex work-related stigma and violence. Addressing the mental health needs of female KPs should be a priority in mental health programs. A study by Kanayama et al.[12] found that female sex workers are at a higher risk of mental health issues due to gender-based violence and discrimination.

Among KPs, FSW had the highest prevalence of psychological distress (12.0%), followed by PWID (8.5%) and MSM (7.5%). Different KP groups may face distinct stressors, such as the stigma associated with sex work for FSW or drug-related challenges for PWID. Tailored mental health support programs should consider the specific needs of each KP subgroup. Research by Shareef [13] highlights the importance of understanding different groups' unique challenges in addressing mental health issues. In addition, participants aged 41-50 had the highest prevalence of psychological distress (14.6%) compared to other age groups. This could be linked to cumulative stressors over time or age-related health concerns. Mental health

interventions for KPs should consider age-related factors in their approach. Studies by Carpenter et al. [14] suggest that older individuals in key populations may have distinct mental health needs due to their life experiences and health challenges.

Furthermore, Akwa Ibom (53.6%) and Lagos state (31.6%) had the highest prevalence of psychological distress among KPs. Regional differences may be attributed to variations in access to healthcare, cultural factors, and socioeconomic disparities. Identifying regions with higher prevalence can guide resource allocation for mental health services. A study by Adesina et al. [15] highlighted regional disparities in mental health outcomes in Nigeria. Also, participants who tested positive for HIV, had a history of substance use, or experienced gender- based violence had higher levels of psychological distress. Co-occurring health and psychological challenges can exacerbate psychological distress among KPs. Integrating mental health support into HIV care, substance use treatment, and gender-based violence prevention programs is crucial. Research by Azrin [16] emphasizes the need for integrated care for persons with multiple health and psychological concerns.

**Association between Sociodemographic Factors and Psychological Distress among KP's enrolled in OSS.**

The study found that female participants had a higher chance of experiencing psychological distress than males. This finding aligns with existing literature that highlights the vulnerability of females to mental health issues due to various sociocultural and biological factors [17,18]. It underscores the importance of gender-sensitive mental health interventions for female KPs within OSS programs. Further analysis revealed that among the KPs, FSWs were 1.519 times more likely, and PWID were 1.953 times more likely to experience psychological distress than MSM. This finding suggests that specific subpopulations within KPs may have distinct mental health needs and risk factors. Similar studies have demonstrated the unique challenges FSW and PWID face regarding mental health [19,20]. Tailoring mental health support to these subgroups is imperative.

Participants aged 20 and below were found to be 4.644 times more likely to experience psychological distress than those aged 50 and above. This result highlights the vulnerability of younger individuals within KPs, which may be attributed to a lack of coping skills, social support, and stigma-related stress [21]. Mental health interventions targeting younger KPs are essential. The study revealed significant regional disparities, with KPs in Akwa Ibom and Lagos states facing significantly elevated odds of experiencing psychological distress.

compared to those in Bayelsa (*P*<0.001). This geographic variation suggests that local contextual factors, healthcare infrastructure, and social support networks may contribute to mental health disparities [22]. Tailored interventions based on regional needs are warranted.

Additionally, participants with a positive HIV status were found to have a higher likelihood of experiencing psychological distress than those without the virus. This counterintuitive result may be attributed to the stigma and psychological stressors associated with HIV diagnosis [23]. Comprehensive HIV care should incorporate mental health support to address this dual burden. Also, participants who engaged in the abuse of alcohol and drug substances were more likely to experience psychological distress compared to those who did not. Additionally, participants with a history of Gender-Based Violence (GBV) were more likely to exhibit psychological distress. These findings underscore the interplay between substance abuse, GBV, and mental health issues among KPs [24]. Integrated interventions addressing both substance abuse and GBV alongside mental health are essential.

**KP's enrolled for MHPSS Intervention.**

The findings indicate that all (100%) participants received a brief intervention, with 73.8% subsequently referred for further psychological intervention. Among those referred, 47.1% were eventually enrolled for comprehensive interventions. The reasons for the observed prevalence of PD enrolment among KPs in OSS are multifaceted. Understanding these factors is essential for tailoring interventions to address MHPSS needs effectively. One key factor contributing to the observed MHPSS enrolment rates may be destigmatization efforts integrated into One-Stop Shop services. Prior studies have emphasized the importance of creating safe, non-judgmental environments for people seeking help in mental health crises. The destigmatization efforts might have encouraged more participants to accept referrals for further psychological intervention. Another contributing factor could be the customization of interventions to meet the unique needs of KPs. Research has shown that tailored interventions are more effective in engaging and retaining marginalized populations in mental health services [25]. OSS may have successfully employed such approaches. Peer support programs within OSS may have played a significant role in encouraging enrolment for MHPSS interventions. Peer support has reduced barriers to mental health service utilization among KPs [26,27].

**Types of intervention received by KP with PD**

The findings indicate that KPs enrolled in the One-Stop Shop program received a range of MHPSS interventions. The most prevalent interventions reported by participants were.

psychoeducation (21.2%), Coping skills training (17.7%) and Motivational enhancement (12.9%). The high prevalence of psychoeducation can be attributed to the effectiveness of this intervention in enhancing awareness and knowledge about mental health issues among KPs. Psychoeducation helps individuals understand their emotions, reduce stigma, and make informed decisions about their mental health. Previous research by Onnela et al. [28] found that psychoeducation significantly improved mental health awareness, leading to increased utilization of mental health services. In addition, coping skills training is essential for KPs who often face discrimination, stigma, and other stressors. This intervention equips them with practical strategies to manage stress, anxiety, and other psychological challenges that may arise due to their marginalized status. A study by Zakiyah and Rofi'ah [29] demonstrated that coping skills training effectively reduced stress and improved mental well-being among marginalized populations. The findings emphasize the importance of addressing stigma related to mental health among KPs. Increased psychoeducation can help reduce stigma and promote a more supportive environment for mental health services. Also, motivational enhancement is critical in engaging KPs in the MHPSS program and motivating them to participate actively. This finding suggests that KPs may require additional motivation and support to access mental health services. Salize et al. [30] conducted a study highlighting the importance of motivational enhancement in engaging hard-to-reach populations, such as KPs, in mental health programs.

**Characteristics of KPs with PD enrolled for interventions.**

The study found that most participants (72.7%) received in-person counselling, indicating a preference for face-to-face interaction. A smaller proportion (24.6%) received virtual counselling, while a minority (2.7%) participated in group sessions within the community. The preference for in-person counselling may be driven by the need for a safe and confidential space to discuss sensitive issues. Virtual counselling may face limitations in terms of privacy and technology access. This distribution may reflect participants' comfort levels with different counselling modalities and access to technology [31]. Also, the study revealed that 74.0% of participants attended only one counselling session.

In contrast, 25.5% attended two sessions, and a minimal 0.5% attended three. The high percentage of participants attending only one session could be due to the perceived effectiveness of a single session or practical constraints, such as work or other commitments. This distribution suggests that most KPs may perceive one session as sufficient for addressing their MHPSS needs, highlighting potential time and resource constraints. In contrast, this

pattern of low utilization may be attributed to various factors, such as time constraints, stigma, and fear of disclosure.

Approximately 68.3% of participants demonstrated good insight into their PD issues, 23.0% exhibited fair insight, and 8.6% lacked insight. Education and awareness programs may influence disparity in insight levels to reduce stigma and increase mental health literacy among KPs. This variation in insight levels may be attributed to factors such as education, awareness, and stigma surrounding mental health, affecting individuals' ability to recognize and acknowledge their concerns. The study found that 86.6% of enrolled KPs experienced mild PD issues, 12.6% of individuals' issues, and only 0.5% reported severe issues. The predominance of mild symptoms may indicate that KPs seek help early, and One-Stop Shop interventions effectively address initial concerns before they escalate. The predominance of mild symptoms suggests that One-Stop Shop interventions may effectively address early-stage PD concerns among KPs. Service providers must assess and address this gap through targeted interventions and psychoeducation [32].

**Limitations of the study**

One limitation of this study is the reliance on clinical data, which may not capture all relevant variables or nuances related to MHPSS among KPs. Since the study used existing data from Heartland Alliance LTD/GTE, it may not have been collected initially to address the specific research questions of this study, potentially limiting the depth of insights and the ability to explore certain factors in greater detail. Additionally, the study's focus on six selected Nigerian states may not fully represent the diversity of PD experiences and needs across the entire country, limiting the generalizability of the findings to the broader Nigerian population.

# CONCLUSION

The study provides valuable insights into the prevalence and factors associated with mental health and psychological support service utilization among KPs enrolled in One-Stop Shop interventions in Nigeria. The findings underscore the critical importance of addressing mental health within the broader context of individual well-being, aligning with the World Health Organization's perspective. Stigma remains a significant barrier to seeking mental health services in Nigeria, emphasizing the need for destigmatization efforts. The study reveals varying levels of psychological distress among KPs, influenced by sociodemographic factors, regional disparities, and unique challenges faced by specific subgroups. These findings

highlight the necessity of tailoring mental health interventions to the specific needs of different KP subpopulations and age groups.

Furthermore, the study demonstrates the success of the One-Stop Shop program in engaging KPs in MHPSS interventions, with a preference for in-person counselling and a majority attending only one session. The predominance of mild symptoms suggests early intervention effectiveness. However, factors contributing to low session attendance warrant further investigation. Overall, this study underscores the urgency of addressing mental health issues among KPs in Nigeria and highlights the importance of tailored, destigmatized, and region- specific interventions to enhance the well-being of this vulnerable population. Future efforts should focus on reducing barriers to access, increasing awareness, and integrating mental health support into existing KP programs.

**Acknowledgments**

A sincere gratitude to Heartland alliance LTD/GTE (HALG) for their invaluable support in facilitating and contributing to this research, their assistance has been instrumental in advancing this study.

**Authors Contribution**

**Conceptualization:** Roger Abang, Bartholomew Ochonye, Olaniyi Felix Sanni, Godwin Emmanuel

**Data curation:** Godwin Emmanuel, Olaniyi Felix Sanni

**Formal analysis:** Olaniyi Felix Sanni, Roger Abang

**Investigation:** Olugbemi Motilewa, Nanribet Mwoltu, Godwin Emmanuel, Roger Abang

**Methodology:** Paul Umoh, Abiye Kalaiwo

**Software:** Olaniyi Felix Sanni

**Supervision:** Olaniyi Felix Sanni, Abiye Kalaiwo, Roger Abang

**Validation:** Olaniyi Felix Sanni, Godwin Emmanuel, Paul Amechi, Paul Umoh

**Writing – original draft:** Paul Amechi, Olaniyi Felix Sanni, Nanribet Mwoltu, Roger Abang

**Writing – review & editing:** Abiye Kalaiwo, Paul Amechi, Nanribet Mwoltu, Paul Umoh

# REFERENCES

1. **Milne A. Mental health, psychological well-being, successful ageing and quality of life. Ment Heal Later Life. 2020;33–52.**
2. **Afe AJ, Onyema M, Ojoye AO, Iwuoha E. Analysis of HIV Treatment Cascade Among Key Affected Population in Lagos, Nigeria. Eur J Med Heal Sci. 2019;1(3):57-69.**
3. **Rayson J, Alba B. Experiences of stigma and discrimination as predictors of mental health help-seeking among sex workers. Sex Relatsh Ther. 2019;34(3):277–89.**
4. **Zahn R, Grosso A, Scheibe A, Bekker LG, Ketende S, Dausab F, et al. Human rights violations among men who have sex with men in Southern Africa: Comparisons between legal contexts. PLoS One. 2016;11(1).**
5. **Abeln B, Love R. Bridging the Gap of Mental Health Inequalities in the Transgender Population: The Role of Nursing Education. Issues Ment Health Nurs. 2019;40(6):482–5.**
6. **Chibanda D. Programmes that bring mental health services to primary care populations in the international setting. Int Rev Psychiatry. 2018;30(6):170–81.**
7. **Arora R. Opinions and Preferences Towards a Community Mental Health Center. Dev Mark Sci Proc Acad Mark Sci. 2015;337–40.**
8. **Carroll JFX, Mcginley JJ. Guidelines for Using the Mental Health Screening Form**

# III. 2000;

9. **WHO. Mental Health [Internet]. World Health Organisation. 2022. Available from: https://**[**www.who.int/health-topics/mental-health#tab=tab_1**](http://www.who.int/health-topics/mental-health#tab%3Dtab_1)
10. **Williams NG and C. Mental Health Crisis in Nigeria: Community Mental Health Care Proposal in the Idiken Slums, Ibadan Oyo State, Nigeria. 2021;**
11. **Viertiö S, Kiviruusu O, Piirtola M, Kaprio J, Korhonen T, Marttunen M, et al. Factors contributing to psychological distress in the working population, with a special reference to gender difference. BMC Public Health. 2021;21(1).**
12. **Kanayama Y, Yamada H, Yoshikawa K, Aung KW. Mental Health Status of Female Sex Workers Exposed to Violence in Yangon, Myanmar. Asia-Pacific J Public Heal. 2022;34(4):354–61.**
13. **Shareef HS. Challenges faced by mental health patients and role of community. Pure Appl Biol. 2021;10(4).**
14. **Carpenter BD, Gatz M, Smyer MA. Mental Health and Aging in the 2020s. Am Psychol. 2022;77(4):538–50.**
15. **Adesina MA, Adesanya T, Olufadewa II. Mental Health and Conflict in Nigeria: An Overview. Eur J Environ Public Heal. 2020;4(1).**
16. **Azrin ST. Integrated care: High-impact mental health-primary care research for patients with multiple comorbidities. Psychiatr Serv. 2014;65(4):406–9.**
17. **Chakraborti S. Women with Mental Illness: A Psychosocial Perspective. Gend Ment Heal Comb Theory Pract. 2020;33–46.**
18. **Steen M, Francisco AA. Maternal mental health and wellbeing. ACTA Paul Enferm. 2019;32(4):III–VI.**
19. **Kaló Z. Women Who Use Drugs and Mental Health. Impact Glob Drug Policy Women Shifting Needle. 2020;67–74.**
20. **Panneh M, Gafos M, Nyariki E, Liku J, Shah P, Wanjiru R, et al. Mental health challenges and perceived risks among female sex Workers in Nairobi, Kenya. BMC Public Health. 2022;22(1):1–14.**
21. **Segrin C, Mcnelis M, Swiatkowski P. Social Skills, Social Support, and Psychological Distress: A Test of the Social Skills Deficit Vulnerability Model. Hum Commun Res. 2016;42(1):122–37.**
22. **Hudson CG. Disparities in the geography of mental health: Implications for social work. Soc Work (United States). 2012;57(2):107–19.**
23. **Littleton T, Choi YJ, McGarity S V. Psychological and social correlates of HIV stigma among people living with HIV. J HIV/AIDS Soc Serv. 2020;19(1):74–89.**
24. **Prestage G, Hammoud M, Jin F, Degenhardt L, Bourne A, Maher L. Mental health, drug use and sexual risk behavior among gay and bisexual men. Int J Drug**

**Policy. 2018;55:169–79.**

25. **Schueller SM, Hunter JF, Figueroa C, Aguilera A. Use of Digital Mental Health for Marginalized and Underserved Populations. Curr Treat Options Psychiatry. 2019;6(3):243–55.**
26. **Davis JK, Pilgrim SI. Maximizing Utilization of Peer Specialists in Community Mental Health: The Next Step in Implementation. J Psychosoc Rehabil Ment Heal. 2015;2(1):67–74.**
27. **Mayor S. Peer support reduces hospital readmissions for mental health crises, shows study. BMJ. 2018;362.**
28. **Onnela A, Hurtig T, Ebeling H. A psychoeducational mental health promotion intervention in a comprehensive schools: Recognizing problems and reducing stigma. Health Educ J. 2021;80(5):554–66.**
29. **Zakiyah A, Rofi’ah IA. Coping and Stress Management Training as an Effort to Improve Stress Adaption Ability. J Kesehat. 2023;14(1):143.**
30. **Salize HJ, Arnold M, Uber E, Hoell A. Verbesserung der psychiatrischen Behandlungsprävalenz bei Risikopersonen vor dem Abrutschen in die Wohnungslosigkeit. Psychiatr Prax. 2017;44(1):21–8.**
31. **Armstrong, Victoria E. Durham E-Theses Mental distress and stigma: exploring the signicance of interactions in the context of support provision. 2016; Available from:** [**http://etheses.dur.ac.uk**](http://etheses.dur.ac.uk/)
32. **Hook K, Ametaj A, Cheng Y, Serba EG, Henderson DC, Hanlon C, et al. Psychotherapy in a Resource-Constrained Setting: Understanding Context for Adapting and Integrating a Brief Psychological Intervention Into Primary Care. Psychotherapy. 2021;58(4):557–75.**

Figures	[Click here to access/download;Figure;Figures.docx](https://www.editorialmanager.com/pone/download.aspx?id=34682484&guid=35bbe246-a5f9-4ec6-b4e9-65fe9b879175&scheme=1)[<https://www.editorialmanager.com/pone/download.aspx?id=34682484&guid=35bbe246-a5f9-4ec6-b4e9-65fe9b879175&scheme=1>](https://www.editorialmanager.com/pone/download.aspx?id=34682484&guid=35bbe246-a5f9-4ec6-b4e9-65fe9b879175&scheme=1)

**Figure Caption**

Figure 1: KP’s enrolled for MHPSS Intervention.

Alt text: Pie chart describing the proportion of Key population who accepted or declined MHPSS

Figure 2: Types of intervention received by KP with PD

ALT Text: A Bar chart consisting of thirteen (13) Intervention services received by Key population with PD

**47.1%, 752**

**52.9%, 843**

**Enrolled for Intervention**

**Declined Intervention**

**Fig 1: KP’s enrolled for MHPSS Intervention**.

21.20%, 473

17.70%, 395

12.90%, 289

12.60%, 281

9.80%, 219

6.40%, 144

4.60%, 102

4.20%, 95

4.00%, 90

1.50%, 34

1.00%, 22

3.90%, 88

**Fig 2: Types of intervention received by KP with PD**

500

450

400

350

300

250

200

150

100

50

0

0.20%, 4

Data file

[Click here to access/download](https://www.editorialmanager.com/pone/download.aspx?id=34682574&guid=52ce944a-1319-4980-8248-acc12e04b6fd&scheme=1)

[Supporting Information 3 MPHSS.xlsb](https://www.editorialmanager.com/pone/download.aspx?id=34682574&guid=52ce944a-1319-4980-8248-acc12e04b6fd&scheme=1)
