# Supplementary material for: Prevalence and factors associated with psychological distress among key populations in Nigeria
Source: PLoS One. 2024 Apr 16;19(4):e0300901. doi: 10.1371/journal.pone.0300901 (PMC11020377; doi:10.1371/journal.pone.0300901)
Supplement: Attachment — Submitted filename: Rebuttal letter.docx [file pone.0300901.s003.docx]

**Rebuttal letter**

| **Review** | **Response** |
| --- | --- |
| **Abstract** | |
| **Review comment** | |
| There was no information about sample size and the type of statistical analysis or test conducted. | A retrospective study was conducted, so there was no need for a sample size. Statistical analysis has been added. |
| Arrange key words in alphabetical order of the first letters and confirm they are available on the MeSH database | Keywords have been updated. |
| The abstract needs to be rewritten to pass across the message in the full manuscript. | Abstract rewritten |
| **Introduction** | |
| **Technical Review** | |
| will be good to provide how metal health challenges impact on KPs overall lifestyle and health outcomes and how findings from this study can help in improving their health. | Individuals with psychological distres**s** in these groups may encounter … |
| **Review comment** | |
| There should be a brief description of One-Stop-Shops' (OSS) as there are many references. | A brief description has been added to the methodology section. |
|  |  |
| **Methodology** | |
| **Technical Review** | |
| - Participants – rephrase the sentences to read like “methods” and not like “results”. | The participants section has been removed. |
| - Study design – rewrite this section by including details about the reason for purposive sampling, sampling technique, sample size e.t.c. | The study design has been rewritten. |
| - Settings – Define what is one-stop-shop, objectives and modus operandi (briefly); provide justifications why the 6 states were selected. Did the 17 OSS comprise all OSS supported by this organization in the 6 states or there are other ones that have not been included? If yes, what are the inclusion/exclusion criteria in selecting the OSS? | The objective and modus operandi have been updated. |
| - Data collection: some components of the study design where the questionnaire was described should be move to this section. Clearly describe how the data was collected – is it using CAPI, self-administered questionnaire e.t.c? The language used in administering the questionnaire, reverse translation (where applicable) e.t.c. This section can be merged with data analysis as Data Collection & Analysis. Also clearly outline variables used in each of the 2 analysis groups (descriptive and association). Define what is PD in the context of analyzed variables. | Section rewritten |
| **Review comment** | |
| The manuscript was well written with both descriptive and inferential statistical analysis done (Chi Squared test and Logistic regression and proper interpretation of the results). However, The data analysis section of the methodology needs to be updated as this statement "Logistic regression analysis was used to determine any correlations between demographics and PD prevalence." is misleading and confusing. The use of the word "association" instead of "correlation" is more appropriate | Done, ‘association’ used instead of correlation |
| Also, the statement "The impact of health measures on PD results was investigated using multiple regression analysis" is too vague. Please provide more information and context to what the heath measures are. | Statement have been deleted. |
| Write OR and AOR in full on their first appearances in the manuscript with the acronym in the parenthesis. Don't assume everybody knows their meaning. Write OR and AOR in full on their first appearances in the manuscript with the acronym in the parenthesis. Don't assume everybody knows their meaning. | Done, Odd ratio and adjusted odd ratio have both been written in full in their first appearance. |
| A methodology section should describe how you collected, organized, and analyse the data. The flow mostly starts with Study design, study population, inclusion and exclusion criteria, sampling methods, definition of variables, statistical analysis etc | Done |
| A result was wrongly presented in the methodology section, it should be reported in the appropriate research language at the result section. | Moved to the result section |
| This language should be modified as there is no such thing as HALG services. There could be PSS or MHPSS services from HALG SDP | Language have been modified |
| This manuscript come across more like a quasi-experimental study than a cross sectional study, the author is advised to decide what the purpose of this study is. | This study takes a mixed-methods approach, combining elements of cross-sectional and quasi-experimental study designs to investigate the prevalence, associated factors, and impact of a Mental Health and Psychosocial Support (MHPSS) intervention on psychological distress among Key Populations in Nigeria. |
| There is the need to mention the ethical clearance obtained for this study. This type of study requires a proper ethical approval from an IRB/REC because of the nature of the investigation and scope (about 6 states) | Ethical clearance was obtained from IREC University of Uyo |
| **Results** | |
| **Technical Review** | |
| - Tables title/captions – indicate time period. |  |
| - Table 1 should indicate socio-demographic characteristics of the participants. Socio-economic factors are critical in PD and must be well analyzed here so that when describing study limitations’ case could be made about potential “confounders”. | Sociodemographic have been added |
| - Also, variables like level of education, occupation are critical. | Unfortunately, education level was not captured.  Also, we only look at their categories (MSM, FSWs and PWIDs) as their major roles in the society instead of any other occupation. For example, most FSWs enrolled in OSS in Nigeria are full time sex workers with no other occupation. |
| - The results section doesn’t flow well with the methods and introduction or the title of the study. Several other things including interventions are included in the results while they were not mentioned as part of the study objectives. The tile should clearly give the reader an idea of what to expect when reading the paper. | The intervention has been included in the study objective |
| **Review comment** | |
| The title of table 1 should be updated to reflect socio demographic characteristics | Table 1 have been updated |
| Why not start the table with socio demographic variables in this order Age group, Sex, Key Population type, State, etc | The suggested order has been followed |
| What has description of intervention got to do with a cross sectional study? | A mixed method study design was used which included both a cross sectional and quasi experimental study design. |
| **Discussion** | |
| **Technical Review** | |
| The discussions require integration. Findings were discussed in “silos” under different subsections. The findings should be linked and integrated and discussed generally as a single study | The study discussion sections have been integrated. |
| **Review comment** | |
| The author should be consistent with the in-text citation style. | Done |
| This manuscript come across more like a quasi-experimental study than a cross sectional study, the author is advised to decide what the purpose of this study is. | This study takes a mixed-methods approach, combining elements of cross-sectional and quasi-experimental study designs |
| Conclusion |  |
|  |  |
| References |  |
